# Mutations in the Non-Structural Protein-Coding Sequence of Protoparvovirus H-1PV Enhance the Fitness of the Virus and Show Key Benefits Regarding the Transduction Efficiency of Derived Vectors

**DOI:** 10.3390/v10040150

**Published:** 2018-03-27

**Authors:** Hamidreza Hashemi, Alexandra-Larisa Condurat, Alexandra Stroh-Dege, Nadine Weiss, Carsten Geiss, Jill Pilet, Carles Cornet Bartolomé, Jean Rommelaere, Nathalie Salomé, Christiane Dinsart

**Affiliations:** 1 Infection, Inflammation and Cancer Program, Tumor Virology Division (F010), German Cancer Research Center (DKFZ), Im Neuenheimer Feld 242, 69120 Heidelberg, Germany; hamidreza.hashemi83@gmail.com (H.H.); a.dege@dkfz-heidelberg.de (A.S.-D.); n-weiss@gmx.net (N.W.); cargeiss@uni-mainz.de (C.G.); jill.pilet@ens.fr (J.P.); carles.cornet.63@gmail.com (C.C.B.); j.rommelaere@dkfz-heidelberg.de (J.R.); 2 Institut National de la Santé et de la Recherche Médicale U701, German Cancer Research Center (DKFZ), 69120 Heidelberg, Germany; nathalie.salome@inserm.fr; 3 INSERM U1109, UDS Institut de Virologie, 3 rue Koeberle, 67091 Strasbourg CEDEX, France

**Keywords:** protoparvovirus H-1PV, fitness mutants, parvoviral vectors

## Abstract

Single nucleotide changes were introduced into the non-structural (NS) coding sequence of the H-1 parvovirus (PV) infectious molecular clone and the corresponding virus stocks produced, thereby generating H1-PM-I, H1-PM-II, H1-PM-III, and H1-DM. The effects of the mutations on viral fitness were analyzed. Because of the overlapping sequences of NS1 and NS2, the mutations affected either NS2 (H1-PM-II, -III) or both NS1 and NS2 proteins (H1-PM-I, H1-DM). Our results show key benefits of PM-I, PM-II, and DM mutations with regard to the fitness of the virus stocks produced. Indeed, these mutants displayed a higher production of infectious virus in different cell cultures and better spreading capacity than the wild-type virus. This correlated with a decreased particle-to-infectivity (P/I) ratio and stimulation of an early step(s) of the viral cycle prior to viral DNA replication, namely, cell binding and internalization. These mutations also enhance the transduction efficiency of H-1PV-based vectors. In contrast, the PM-III mutation, which affects NS2 at a position downstream of the sequence deleted in Del H-1PV, impaired virus replication and spreading. We hypothesize that the NS2 protein—modified in H1-PM-I, H1-PM-II, and H1-DM—may result in the stimulation of some maturation step(s) of the capsid and facilitate virus entry into subsequently infected cells.

## 1. Introduction

The rat parvovirus H-1PV belongs to the genus *Protoparvovirus* within the subfamily *Parvovirinae*. These autonomous parvoviruses are non-enveloped viruses that consist of an icosahedral capsid containing a single-stranded DNA of about 5000 nucleotides. The parvoviral genome consists of two overlapping transcription units whose expression is under the control of two promoters [1]. The P4 promoter drives the expression of the non-structural proteins (NS1 and NS2) and the P38 promoter controls the expression of the capsid proteins (VP1 and VP2) [2] and of the non-structural small alternatively translated (SAT) protein [3]. The NS1 protein is a mainly nuclear, phosphorylated, multifunctional protein of about 83 kDa that is required for the replication of the viral genome and transactivation of the P38 promoter. It plays a role in the egress of progeny virions and is the main effector of parvoviral cytotoxicity [4,5,6]. The small NS2 proteins (about 25 kDa) of the protoparvovirus minute virus of mice (MVM) consist of three isoforms that are generated by alternative splicing and differ in their carboxyl termini [7]. NS2 is essential for various steps of the parvovirus life cycle: it has been reported to play a role during viral DNA replication [8,9,10], translation of viral mRNA [11,12], capsid assembly [9], and parvoviral cytotoxicity [13]. Yet, the specific NS2 function(s) is (are) still unclear. NS2 is predominantly located in the cytoplasm and only non-phosphorylated NS2 can be detected in the nuclei of infected cells [7]. NS2 interacts with members of the 14-3-3 protein family [14], the survival motor neuron (SMN) protein [15,16], and the nuclear export factor chromosomal region maintenance protein-1 (CRM1) [17]. A supraphysiological nuclear export signal in NS2 interacts with and sequesters CRM1 in the cytoplasm at the nuclear periphery in infected cells. MVM mutants with disabled NS2-CRM1 interaction fail to be exported to the cytoplasm and display dramatically reduced fitness, supporting a key role of NS2/CRM1 interaction in the control of maturation and the egress of viral particles [18,19,20]. Our laboratory previously isolated and analyzed a fitness mutant of H-1PV (Del H-1PV) that exhibits an in-frame deletion in the NS protein-coding sequence [21,22]. The 114-nucleotide-long deletion in the genome of this mutant showed key benefits with regard to the fitness of Del H-1PV compared with wild type (wt) H-1PV release and particle-to-infectivity (P/I) ratio *in vitro* and their tumor suppressive activity *in vivo*. The increased infectivity correlated with an accelerated egress of Del H-1PV progeny virions and with the stimulation of some step(s) of the viral cycle, in particular improved virus uptake. We hypothesized that the shortened NS2 and/or NS1 products expressed by Del H-1PV may be more efficient than the full-length proteins at driving egress maturation step(s) (posttranslational modifications) of the capsid proteins, leading to stimulation of virus nuclear export and fitness of the virus produced [22]. The deletion in Del H-1PV affects not only NS2 but also NS1. Since NS1 is a multifunctional protein required for several steps of the viral cycle it raises the possibility that it may also contribute to the fitness phenotype of Del H-1PV. To address this question, point mutations affecting NS1 and NS2 or NS2 alone were introduced in the H-1PV genome in the region of the 114-nucleotide-long deletion of Del H-1PV. These mutations were chosen based on a study on the closely related parvovirus MVMi [23]. In this study, MVM fitness variants were selected in mice with severe combined immunodeficiency (SCID) by passive immunotherapy with a neutralizing monoclonal antibody recognizing the MVMi capsids. Surprisingly some of those mutants harbored amino acid changes close to the CRM1 binding site of the NS2 protein. These mutants, having a few mutations close to the corresponding deleted region of Del H-1PV, exhibit an enhanced cytoplasmic sequestration of CRM1, suggesting that mutated NS2 proteins with higher affinity for CRM1 may increase parvovirus fitness [23]. This prompted us to introduce the mutations described for MVMi into the genome of H-1PV—generating H1-PM-I, H1-PM-II, H1-PM-III, and a double mutant H1-DM harboring both PM-I and PM-II mutations—and to analyze their effect on viral fitness. While H1-PM-I, H1-PM-II, and H1-DM clearly showed enhanced fitness in human cells compared with wild type, as indicated by their low particle-to-infectivity ratios and higher spreading capacity, H1-PM-III, which contains one amino acid substitution (L153M) in NS2 located slightly downstream of the sequence deleted in Del H-1PV, was dramatically impaired in its replication and spreading. 

Recently, in a first phase I/IIa clinical trial, wild-type H-1PV was administered to recurrent glioblastoma patients [24] and provided promising results for further clinical investigation in cancer patients. However, the efficacy of H-1PV might be limited by the fact that the rodent protoparvovirus does not usually propagate in human tumor cells. Consequently, optimization of such viral therapeutics is needed. Our results indicate that the mutations introduced to generate H1-PM-I, H1-PM-II, and H1-DM and their derived vectors show key benefits for the virions produced regarding their enhanced infectivity and transduction efficiency and may provide alternative tools for their application in cancers where wild-type H-1PV is less efficient.

## 2. Materials and Methods

### 2.1. Infectious Molecular Plasmids

Point mutations (see Table 1) were introduced into the infectious molecular clone of wild-type H-1PV (pH1) [24] using the Quick Change Site-Directed Mutagenesis Kit (Qiagen, Hilden, Germany) to generate pH1-PMI, pH1-PMII, and pH1-PMIII. Specific PCR amplifications were performed in 50 µL reaction buffer containing 50 ng of pH1 template plasmid DNA, 125 ng of each forward and reverse primer (GATC Biotech, Konstanz, Germany), 200 µM of each dNTP, and 2.5 units of high fidelity DNA polymerase (PfuUltra HF, Agilent Technologies, Santa Clara, CA, USA). The PCR cycles were performed as following: 95 °C for 55 s for initial denaturation; 12 cycles of 95 °C for 30 s and 55 °C for 60 s for annealing; and 68 °C for 8 min for extension. To generate the double mutant, the PMI primers were used to introduce the PMI mutation into pH1-PMII. All constructs were verified by sequencing (GATC Biotech, Konstanz, Germany).

### 2.2. H-1PV-Based Vectors

The H-1PV-based vectors pChi-H1/Δ800 and pChi-H1/Gluc have been described elsewhere [25]. The EcoRI-HindIII restriction DNA fragments from pH1-PMI, pH1-PMII, pH1-PMIII, and pH1-DM were cloned into the vectors, generating pChi-H1-PMI/Δ800, pChi-H1-PMII/Δ800, pChi-H1-PMIII/Δ800, and pChi-H1-DM/Δ800 and pChi-H1-PMI/GLuc, pChi-H1-PMII/GLuc, pChi-H1-PMIII/GLuc, and pChi-H1-DM/GLuc, respectively.

### 2.3. Construction of H-1PV Split Vectors

P4-NS and P38-VP cassettes were constructed in split vectors as follows: The P4-NS and P38-VP regions of pH1, pH1-PMIII, and pH1-DM were PCR amplified using a high-fidelity DNA polymerase (Q5^®^ DNA polymerase, NEB) with the following primer sets: P4-NS (forward: 5′-GTTCTACTCGAGATAAGCGGTTCAGAGAGTTTGAAACCAAG-3′; reverse 5′-AATAAAGCGGCCGCTCAAGGCTGTTCCCTGGTC-3′); P38-VP (forward: 5′-TATTAACTCGAGCATTACCGTGGTTAGAATAGGCTGTG-3′; reverse: 5′-ATTAAAGCGGCCGCTTAGTATGTCATGTGAGGCACAG-3′). The DNA fragments were digested by XhoI and NotI (sites underlined) and cloned into the pMCS-Gaussia, a promoter-less vector (Life Technologies, Carlsbad, CA, USA), in replacement of the Gaussia luciferase cDNA, resulting in pP4-NS, pP4-NS-PMIII, and pP4-NS-DM and pP38-VP, pP38-VP-PMIII, and pP38-VP-DM, respectively. For reporter gene assays, a 555-bp DNA fragment spanning the P38 promoter of pH1 and pH1-PMIII was PCR amplified using the forward primer 5′-TATTAACTCGAGCATTACCGTGGTTAGAATAGGCTGTG-3′ and reverse primer 5′-TATATTGGATCCTAGTCCAAGGTCAGCTCCTCG-3′. After digestion with the restriction enzymes XhoI and BamHI (sites underlined), the amplified DNA sequences were cloned into pMCS-Gaussia to generate pP38-Gluc and pP38-PMIII-Gluc.

### 2.4. Cell Cultures

Simian virus 40 (SV40)-transformed human newborn kidney (NB-324k) cells [26] were propagated in Eagle’s minimal essential medium (MEM; Sigma-Aldrich, St. Louis, MO, USA) supplemented with 5% fetal bovine serum (FBS, Gibco Life Technologies, Carlsbad, CA, USA), 2mM l-glutamine, and antibiotics (100 U/mL of penicillin G and 100 µg/mL of streptomycin sulfate; Invitrogen). The human embryonic kidney 293T/17, cervix carcinoma HeLa, pancreatic ductal carcinoma Panc-1 cell lines, and the rat glioblastoma cell line RG-2 (American Type Culture Collection) were grown in Dulbecco’s modified Eagle’s medium (DMEM; Sigma-Aldrich, St. Louis, MO, USA) supplemented with 10% FBS, 2 mM l-glutamine, and antibiotics (100 U/mL of penicillin and 100 µg/mL of streptomycin sulfate).

### 2.5. Transfection Assays

293T cells were transfected using calcium phosphate (Sigma-Aldrich, St. Louis, MO, USA) precipitation method as described before [22]. Briefly, a mixture containing 15 µg plasmid DNA and 250 mM CaCl_2_ was incubate with an equal volume of 2X Hanks’ balanced salt solution (HBSS) at room temperature for 20 min. The DNA-CaPO_4_ precipitate was added to 5 × 10^6^ cells seeded in 15 cm plates, which were then incubated at 37 °C and 5% CO_2_. NB-324K cells were transfected with Lipofectamine 3000 (LFA™ 3000, Life Technologies, Carlsbad, CA, USA). Briefly, DNA was diluted in serum-free Opti-MEM™ medium and added to a Lipofectamine 3000 solution according to the manufacturer’s instructions. The mixture was incubated at room temperature for 10 min and then added dropwise to the cell culture medium of NB-324K cells. At 5 h post-transfection, the culture medium was replaced by fresh complete cell culture medium. For the co-transfection experiments with the split vectors, 2.7 × 10^5^ NB-234K cells seeded in 6-well plates (Greiner Bio-One, Solingen, Germany) were transfected with a mixture of pP4-NS (0.5 µg/well) and pP38-VP or pP38-Gluc plasmids (2 μg/well). Alternatively, 1 × 10^5^ NB-324K cells were seeded in 12-well plates and transfected with a mixture of pP4-NS (195 ng/well) and pP38-Gluc plasmid (780 ng/well) and 25 ng/well of a plasmid encoding the secreted luciferase, Cypridina luciferase (pCMV-Cypridina luc, Life Technologies, Carlsbad, CA, USA), to normalize for transfection efficiencies. 

### 2.6. Dual Luciferase Assay

The H-1PV-based split vectors were co-transfected with pP38-Gluc and pCMV-Cypridina luc into NB-324K cells as described above. At various times post-transfection, the cell culture medium was collected and stored at −80 °C until measurement. Both luciferase activities were determined in a white 96-well plate (Greiner Bio-One, Solingen, Germany) containing 25 μL of cell medium and 80 µL of the substrate solution (1 μM Coelenterazine for Gaussia and 1 μM Vargulin for Cypridina) after 1 min incubation at room temperature, using a luminometer (PerkinElmer, Solingen, Germany). The values of the Gaussia luciferase activity were normalized to those of the Cypridina activity.

### 2.7. Virus Production

The mutants and H-1PV were primarily produced by transfection of 293T cells with appropriate plasmid constructs and subsequently amplified by infection of NB-324k cells at a multiplicity of infection (MOI) of 3 × 10^−3^ plaque forming units (PFU)/cell. The recombinant virus stocks were produced as previously described [27]. Cells were harvested 3 days post-transfection or 4–5 days post-infection and lysed by means of three freeze-thaw cycles in VTE buffer (50 mM Tris pH 8.7, 0.5 mM EDTA). The cell lysates were clarified by centrifugation and virus stocks were purified by iodixanol step gradient centrifugation as described previously [28] and titrated as below. 

### 2.8. Titration of Virus Stocks

Infectious virus stocks were titrated on NB-324k cells by plaque assay or by infected cell hybridization assay [27]. Virus titers are expressed as plaque forming units (PFU) or replication units (RU) per milliliter of virus suspension. Full viral particles were quantified either by dot blot hybridization assays [29] or by quantitative real-time PCR. Briefly, viral DNA was extracted from iodixanol-purified virus stocks or subcellular fractions using QIAamp MinElute virus spin kit (Qiagen, Hilden, Germany) as recommended by the manufacturer. The DNA was eluted in nuclease-free water (Life Technologies, Carlsbad, CA, USA) and stored at −20 °C until measurement. A linearized pH1 plasmid in serial dilutions in nuclease-free water was used to standardize the qPCR. Quantification of viral DNA was carried out by real-time qPCR in a volume of 20 µL using TaqMan^®^ Universal Master Mix (Life Technologies, Carlsbad, CA, USA) supplemented with 0.3 µM of NS-specific primers and a dual-labeled TaqMan^®^ probe (5′-6-FAM and 3′-MGB, Europhins MWG, Ebersberg, Germany). PCR cycles were performed in white 96-well plates (Hard-Shell^®^ PCR plates, Bio-Rad, Hercules, CA, USA) for 40 cycles using a qPCR thermocycler (CFX96 Touch™ Real-Time PCR, Bio-Rad). Virus titers are expressed as the number of viral genomes (Vg) per milliliter of virus suspension.

### 2.9. Analysis of Plaque Size

For the size of the plaques and their frequency of occurrence, plaque assays were performed as previously described [22]. Briefly, 3 × 10^6^ NB-324K cells on 15 cm plates (Greiner Bio-One, Germany) were infected with dilutions of wt H-1PV or mutant viruses in 2.5 mL of serum-free MEM (Sigma-Aldrich, Germany) for 1 h at 37 °C. After infection, 20 mL of overlay medium was added to each dish and incubated for 5 days. Living cells were then stained with neutral red-containing staining solution. The number and size of the plaques were measured using the Java-based image processing software ImageJ, version 1.51w (National Institutes of Health, Bethesda, MD, USA).

### 2.10. Immunoblotting

NB-324k cells (2 × 10^5^) were infected with H-1PV or with the various mutants either with a MOI of 3 PFU/cell or with 2225 Vg/cell. For some experiments, 1 × 10^6^ NB-324k cells were transfected with 6 µg of pH1, pH1-PMI, pH1-PMII, pH1-PMIII, and pH1-DM. Cells were harvested at 16 and 20 h post-infection (p.i.) or post-transfection (p.t.) and lysed in RIPA buffer (150 mM NaCl, 10 mM Tris pH 7.5, 1 mM EDTA pH 8.0, 1% NP-40, 0.5% sodium deoxycholate, 0.1% sodium dodecyl sulfate (SDS)) supplemented with a protease inhibitor cocktail (Roche, Penzberg, Germany). After protein quantification (bicinchoninic acid (BCA) protein assay, ThermoScientific, Waltham, MA, USA), 10 µg or 20 µg of total proteins were separated by 10% SDS-polyacrylamide gel electrophoresis (PAGE) and electrotransferred to Protan nitrocellulose membranes (PerkinElmer Life Sciences, Waltham, MA, USA). The membranes were then incubated with rabbit polyclonal antisera directed against either MVMp NS1 or NS2p (α-NS2p) [30] or H-1PV capsid proteins (α-VP) [30] and with appropriate secondary horseradish peroxidase-coupled antibodies (Santa-Cruz Biotech, Santa Cruz, CA, USA). Immunoreactive proteins were revealed by enhanced chemiluminescence (Western Lightning^®^ Plus-ECL, PerkinElmer, Waltham, MA, USA). Images were captured using an ECL imaging system (INTAS Science Imaging, Göttingen, Germany) and protein band densities were quantified with Lab1D image analysis software (INTAS Science Imaging, Germany).

### 2.11. Southern Blot Analysis

Low molecular weight viral DNA was extracted from infected or transfected cells using the DNeasy Blood and Tissue Kit (Qiagen, Hilden, Germany) according to the manufacturer’s instructions. After transfection, low molecular weight DNA was digested with DpnI to selectively degrade the transfected plasmid DNA. Resistance to DpnI cleavage is indicative of DNA replication in eukaryotic cells. Viral DNA replicative forms were separated by 1% agarose gel electrophoresis and processed as previously described [22]. After transfer to nitrocellulose membrane, the DNA was linked to the membrane by heat treatment for 2 h at 80 °C, the membrane was hybridized with a P32-labeled DNA fragment specific for NS, and exposed to autoradiographic film (bioMax MS film; Kodak, Rochester, NY, USA) at −80 °C.

### 2.12. Northern Blot Analysis

Total RNA was extracted from 5 × 10^5^ cells (MOI: 3 PFU/cell) using the RNeasy kit (Qiagen, Hilden, Germany). RNA was assessed for its quality by 1% agarose gel electrophoresis and for its quantity by spectrophotometric measurement. After treatment with RNase-free DNase-1 (NEB), the RNA was fractionated by electrophoresis on a 1% agarose-formaldehyde gel. After transfer under high salt conditions to a Hybond-N+ nylon filter (Amersham, London, UK), the samples were prehybridized with salmon sperm DNA (100 μg/ml) and hybridized overnight at 42 °C with a ^32^P-labeled randomly primed specific VP DNA probe in the presence of 50% formamide and 5% dextran sulfate (Northern Max kit, Life Technologies, Carlsbad, CA, USA). Membranes were then washed under highly stringent conditions and autoradiographed. 

### 2.13. Analysis of Virus Binding

NB-324K cells (5 × 10^5^) were infected at 4 °C for 1 h with wt H-1PV or mutant virus at an MOI of 2225 Vg/mL. Cell culture medium (serum-free MEM) and cells were harvested by centrifugation and suspended in 200 µL of ice-cold phosphate-buffered saline (PBS) and lysed to measure cell-bound virus particles [22]. Cell culture medium was saved to measure free particles. Viral DNA was recovered from both cell lysates and cell culture media using QIAamp MinElute virus spin and DNeasy Blood and Tissue Kit (Qiagen, Hilden, Germany) as recommended by the manufacturer. Viral genome content of the samples was quantified by quantitative real-time PCR (qPCR) as described above. 

### 2.14. Virus Uptake

NB-324k cells (5 × 10^5^) were infected at 4 °C for 1 h with wt H-1PV or the mutants and further incubated at 37 °C. At various times post-infection, cells were washed with PBS and membrane-bound virus particles were removed by treatment for 5 min with 500 μL of trypsin-EDTA solution (0.25% trypsin, 1 mM EDTA, Life Technologies, Carlsbad, CA, USA). The reaction was stopped by adding complete MEM (5% FBS) and cells were collected by centrifugation. Viral genomes were recovered from cell lysates and quantified by qPCR.

### 2.15. Nuclear and Cytoplasmic Fractionation

NB-324K cells were infected as described for virus uptake. At various times post-infection, cells were harvested and a nucleocytoplasmic fractionation was performed using an NE-PER extraction reagents kit (ThermoScientific, Waltham, MA, USA) according to the manufacturer’s instructions. The number of full viral particles was determined in each fraction by real-time qPCR.

## 3. Results

### 3.1. Production and Infectivity of H-1PV Mutants

To determine whether NS1 and/or NS2 was mainly responsible for the fitness of Del H-1PV, which contains an in-frame deletion in the NS coding sequence [22], point mutations were introduced in the corresponding NS sequence of the infectious molecular clone of H-1PV and the virus stocks were produced as described under the Materials and Methods section. For this, pH1-PMI, pH1-PMII, pH1-PMIII, pH1-DM, and the parental pH1 molecular clones were transfected into human 293T cells to generate the master virus stocks of H1-PM-I, H1-PM-II, H1-PM-III, H1-DM, and H-1PV, respectively, and amplified by infection of NB-324k cells. The nucleotide changes in H-1PV genome and the resulting modifications in NS1 and/or NS2 proteins are described in Table 1 and Figure S1. The titers of genome-containing (full particles, in Vg/mL) and plaque-forming (infectious particles, in PFU/mL) viral particles and the corresponding particle-to-infectivity ratios were determined in virus stocks produced from independent productions. 

**Figure 1:**
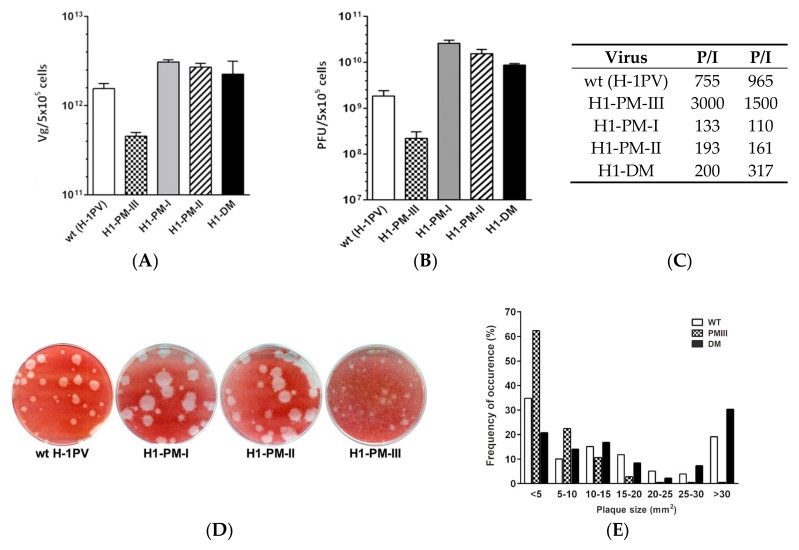
Enhanced infectivity and spreading capacity of H1-PM-I, -PM-II, and –DM, but not of H1-PM-III, compared to wild-type H-1PV in NB-324k cells. (**A**) Progeny virus production infectivity in human NB-324k cells. NB-324k cells infected with wild-type (wt) H-1PV, H1-PM-I, H1-PM-II, H1-PM-III, or H1-DM (MOI, 3 × 10^-3^ PFU/cell) were harvested at day 5 post-infection. Titers of full particles (given in Vg/mL) (**A**) and infectious virus (in PFU/mL) (**B**) were determined after virus purification by qPCR and plaque assay, respectively. The particle-to-infectivity ratio (P/I) (**C**) of the virus stocks is indicated. Results are given from two independent experiments. (**D**,**E**) Plaque size and frequency of occurrence after infection of NB-324K cells. Plaque assays were performed on wt, H1-PM-I-, H1-PM-II- (**D**) or wt, H1-PM-III-, and H1-DM-infected NB-324k cells (**D**,**E**) and the plaque sizes of wt, H1-PM-III-, and H1-DM-infected cells (**E**) were determined from scanned pictures of the plates using ImageJ software. Plaque sizes are given in mm^2^ and frequencies of occurrence are expressed as a percentage of total number of plaques analyzed; 133 ≤ *n* ≥ 201.

Figure 1 shows the results of two virus productions performed in parallel. As indicated in Figure 1A, the titers of full particles (Vg) produced by H1-PM-I, H1-PM-II, and H1-DM infections were only slightly higher than with H-1PV. However, the production of infectious particles (PFU) by the mutants was consistently higher than the H-1PV production (Figure 1B), resulting in a lower P/I ratio of these mutants (Figure 1C)—as previously observed after infection with Del H-1PV [22]—and indicates an enhanced fitness. Surprisingly, the titers of full and, more strikingly, of infectious particles produced by infection with H1-PM-III were reproducibly lower than those obtained with H-1PV, resulting in a higher P/I ratio than H-1PV (Figure 1C).

These results prompted us to examine the spreading of the mutants in comparison with wt H-1PV. For this, plaque assays were performed on NB-324k cells and the size and occurrence frequency of the plaques produced were measured by the image-processing software ImageJ. The occurrence frequency of the plaque sizes produced by each virus was determined and expressed as the percentage of the total number of analyzed plaques. All viruses, including wt H-1PV, produced a mixture of small and large plaques (Figure 1D). However, infection with H1-PM-I, H1-PM-II, and H1-DM gave rise to a higher frequency of large plaques, as shown for H1-DM (Figure 1E). The infection with H1-PM-III resulted in dramatically smaller plaques from which 60% developed plaques whose sizes were less than 5 mm^2^ (Figure 1E). The more efficient propagation of H1-PM-I, -PM-II, and –DM compared with the wild-type H-1PV indicates an enhanced fitness of these mutants, while H1-PM-III shows an attenuated phenotype.

**Figure 2:**
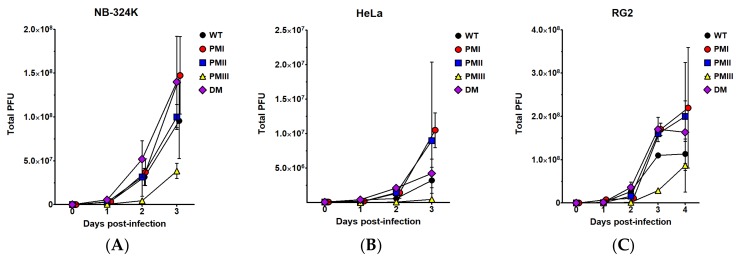
Enhanced infectious progeny production of H1-PM-I, H1-PM-II, and H1-DM in human but not in rat cell lines. Infectious particles of wt H-1PV and derived mutants were measured after infection (MOI, 0.5 PFU/cell) of (**A**) human NB-324k; (**B**) HeLa; and (**C**) rat RG-2 cell lines. The production of infectious particles (total PFU) was determined by plaque assays at the indicated times post-infection (p.i.) for the three cell lines. Results are given as the mean ± SD of plaque numbers measured in duplicate from ≥3 independent experiments.

We next wanted to determine (i) whether the production of infectious particles harboring the mutations was restricted to the human, SV40-transformed, NB-324k cells; and (ii) whether they could also be produced in rat cells since the mutations modify the amino acid composition of the small viral protein NS2, which is dispensable for the replication of H-1PV in human cells but is required in its host cells [9,31]. For this, NB-324k, human cervix carcinoma HeLa cells, and rat glioma RG-2 cells were infected at an MOI of 0.5 PFU/cell with H-1PV or the mutants and virus particles were recovered at various days post-infection and quantified by plaque assays. Figure 2 shows the yields of infectious particles recovered at various days post-infection. As can be seen in Figure 2B, all the virus stocks produced in the HeLa cells, including wt, were lower than in NB-324k cells (Figure 2A). However, HeLa cells yielded higher levels of the mutants H1-PM-I, H1-PM-II, and H1-DM than wild-type (wt) H-1PV, indicating that their fitness was not restricted to NB-324k cells. The production of H1-PM-III in HeLa cells was marginal, yielding only 3-fold higher viruses than the virus inoculum. Surprisingly, an adaptation of the mutants seems to be specific for human cells as it was not seen in rat cells. Indeed, the yields of infectious particles after infection of rat RG-2 cells with H-1PV or the mutants were not significantly different with a 2-fold increase at day 4 p.i. (H1-PM-I) as the highest (Figure 2C). These data show that the mutants were still able to propagate in host rat cells but their endowed fitness capacity adapted to human cells. As it was also observed after infection of NB-324k and HeLa cells, infection of RG-2 cells with H1-PM-III produced the lowest amounts of progeny viruses at any time post-infection (Figure 2C). 

**Figure 3:**
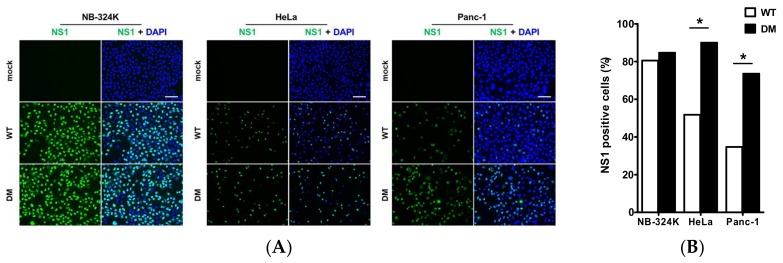
Increased efficiency of infection of H1-DM in various human cell lines. (**A**) Indirect immunofluorescence of NS1 after infection of NB-324k, HeLa, and Panc-1 cells. Cells (4 × 10^3^) were mock-treated or infected with wt H1PV or H1-DM (MOI, 10 PFU/cell) and analyzed at 24 h p.i by indirect immunofluorescence assay for NS1 (green). Nuclei were visualized by co-staining with DAPI (blue) in merge panels, 20x magnification, scale bar = 100 µm; (**B**) Percentage of NS1-positive cells after infection with wt H-1PV or H1-DM. The percentage of NS1-postive cells in (**A**) was determined by counting at least 200 cells in three distinct fields per sample (* *p* < 0.05).

The infectivity of the fitness mutants was further examined by indirect immunofluorescence after infection of NB-324k, HeLa, and human pancreatic adenocarcinoma Panc-1 cells. Cell cultures were virus infected (MOI = 10 PFU/cell) and incubated with a monoclonal specific anti-NS1 antibody 24 h post-infection (Figure 3A). NS1 is known to be a marker of the onset of viral DNA amplification [4]; its increased expression indicates stimulation of the early steps of the viral cycle. NS1-positive cells were expressed as a percentage of DAPI-stained cells examined in the same fields (Figure 3B). Figure 3 shows the results obtained after infection with H1-DM, as a representative mutant for this analysis, and wt H-1PV. As seen in Figure 3, the percentage of NS1-positive cells increased to a maximum in the three cell lines after infection with H1-DM, reaching 85% (NB-324k), 90% (HeLa), and 74% (Panc-1) of total cells, respectively. However, while the percentage of NS1-positive cells only slightly increased in H1-DM compared to wt H-1PV-infected NB-324k cells (5% higher), in HeLa and Panc-1 cells the percentage of fluorescent cells was significantly higher in H1-DM compared to wt H-1PV-infected cells (40% and 39% higher). This indicates that in at least some tumor cells, the mutants have a selective advantage and are worth considering as a more efficient tool to combat tumor cells in which wt H-1PV is not efficient enough. The increased fraction of NS1-positive cells in H1-DM-infected cells compared with wt H-1PV shows that the adaptation correlates with stimulation of an early step(s) of the viral cycle.

### 3.2. Enhanced Transduction Efficiency of H1-PM-I, H1-PM-II, and H1-DM-Based Vectors

**Figure 4:**
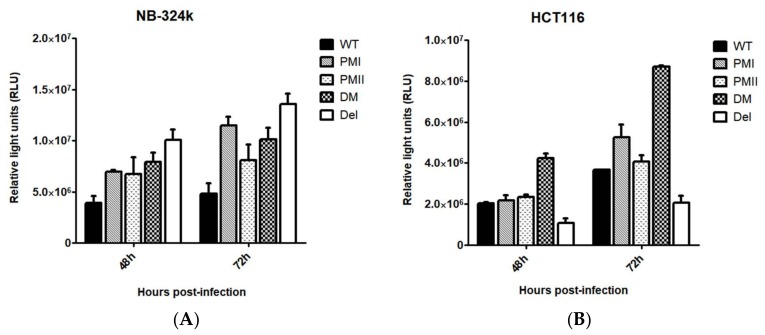
Quantification of luciferase transduction in human NB-324k and HCT-116 cells infected with modified recombinant H-1PV-based vectors. Using 3 × 10^5^ cells, NB-324k (**A**) and HCT-116 (**B**) cells were infected with Chi-H1/Gluc, Chi-H1-PM-I/Gluc, Chi-H1-PM-II/Gluc, Chi-H1-DM/Gluc, and Chi-Del H1/Gluc (MOI, 3 RU/cell). At 48 and 72 h post-infection, Gaussia luciferase activity was determined in the cell culture medium and expressed as relative units. Values are the mean ± SD of three measurements from duplicates.

We reasoned that the mutations in the genome of H1-PM-I, H1-PM-II, and H1-DM might also be beneficial for the production and transduction efficacy of H-1PV-based vectors. Therefore, the mutations (see Table 1) were introduced into the recombinant viral genome (pChi-H1/Gluc) in which the capsid genes (VP) were replaced by the reporter gene Gaussia luciferase (Gluc) [25] and recombinant viruses produced as previously described [27]. Chi-Del H1/Gluc carrying the 114 nt-long deletion of Del H-1PV [22] was also produced for comparison. The transduction efficiency was determined in NB-324k and human colon carcinoma HCT-116 cells infected with the recombinant vectors at an MOI of three replication units (RU)/cell. Figure 4 shows the Gaussia luciferase activity in the cell medium 48 h and 72 h after infection, expressed as relative units. The luciferase activity was higher after transduction with all the mutants than with wild-type Chi-H1/Gluc, irrespective of the cell line and the time post-infection (Figure 4A,B). In NB-324k cells, 72 h post-infection the relative luciferase units were 2- to 3-fold higher after infection with the mutants than with wt Chi-H1/Gluc. The transduced luciferase activity was not significantly different among all the mutants in this cell line (Figure 4A). In HCT-116 cells, the transduction efficiency was significantly higher only after infection with Chi-H1-DM/Gluc, while the transduced luciferase activity was either similar (Chi-H1-PM-I/Gluc and Chi-H1-PM-II/Gluc) or even reduced (Chi-Del H1/Gluc) compared to the wild type (Figure 4B). Taken together, this shows key benefits of all the vectors, including Chi-Del H1/Gluc, with regard to the transduction efficiency in NB-324k cells and of Chi-H1-DM/Gluc in HCT116 cells and confirms that the choice of the modified vector depends on the cell type or origin of the tissue.

### 3.3. Enhanced Viral Protein Accumulation after Infection with the Fitness Mutants but Reduced Levels of Capsid Proteins with H1-PM-III 

**Figure 5:**
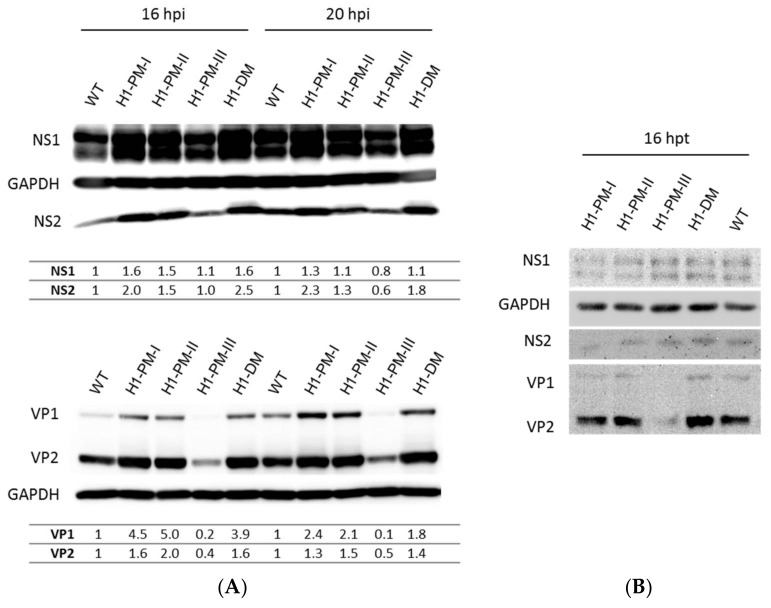
Accumulation of viral proteins expressed from wt H-1PV or the mutants in NB-324k cells. (**A**) Accumulation of viral proteins after infection of NB-324k cells. NB-324K cells (5 × 10^5^) were infected with wt H-1PV, H1-PM-I, H1-PM-II, H1-PM-III, or H1-DM (MOI, 2225 Vg/cell) and further incubated in the presence of neutralizing antibodies. Protein extracts were prepared 16 h and 20 h post-infection and subjected to a 10% SDS-PAGE gel electrophoresis, followed by immunoblotting using rabbit polyclonal antisera recognizing NS1 and NS2 or VP proteins. Signal intensities of the viral proteins were quantified by Lab-1D software and normalized relative to the density of the corresponding wt NS and VP proteins; (**B**) Accumulation of NS1/NS2 and VP proteins expressed from pH1 or mutant-derived plasmids after transfection. NB-324K cells (5 × 10^5^) were transfected with 6 µg of pH1, pH1-PM-I, pH1-PM-II, pH1-PM-III, or pH1-DM protein extracts prepared 16 h post-transfection and processed as for (**A**).

We next wanted to analyze viral protein accumulation after infection of NB-324k cells with H-1PV mutants. For this, NB-324k cells were infected at an MOI of 2225 Vg/cell with H-1PV or each of the H-1PV mutants and the viral proteins were analyzed from the cell lysates by Western blotting 16 and 20 h post-infection. Neutralizing antibodies were added after 2 h of infection to prevent secondary infection cycles and the intensity of the bands was quantified relative to cellular GAPDH, used as a loading control. The intensity of NS1/2 and VP1/2 bands from wt H-1PV was referred to as 1. As shown in Figure 5A, both nonstructural (NS1/NS2, upper panel) and structural (VP1/VP2, lower panel) viral proteins accumulated at higher levels after infection with fitness mutants compared to wt H-1PV. Yet, the increased accumulation of NS1 was moderate: 1.6-fold increase for H1-PM-I and H1-DM and 1.5-fold increase for H1-PM-II 16 h post-infection. At this time, NS2 accumulated up to 2.5-fold more protein after infection with H-1-DM than with wt H-1PV. The enhanced accumulation of VP1/2 was more striking after infection with H1-PM-I, H1-PM-II, and H1-DM than with H-1PV, with up to a 5-fold increase of VP1 (H1-PM-II infection). In contrast to infection data, no significant increase in the levels of NS or VP proteins was observed after transfection with the corresponding plasmids pH1-PM-I, pH1-PM-II, and pH1-DM compared with wt pH1 (Figure 5B), in agreement with the above conclusion that early steps of the viral cycle are stimulated. Interestingly, although the levels of NS1/ NS2 produced after infection with H1-PM-III were similar to those of wt H-1PV, the accumulation of the capsid proteins VP1/VP2 by H1-PM-III was dramatically reduced. This indicates that the attenuated phenotype displayed by H1-PM-III may rely on low levels of VP proteins expressed by infection with this mutant. Whilst NS1/2 levels were similar to those obtained after transfection with pH1, VP1/2 protein accumulation after transfection with pH1-PM-III was strongly decreased (Figure 5B). This indicates that a post-entry step(s), a translation/post-translational step, and/or stability of the proteins may be responsible for the low levels of VP proteins expressed by infection with H1-PM-III.

### 3.4. Enhanced Accumulation of Viral ssDNA after Infection with Fitness Mutants, but Severe Decrease of ssDNA Production in H1-PM-III-Infected Cells

**Figure 6:**
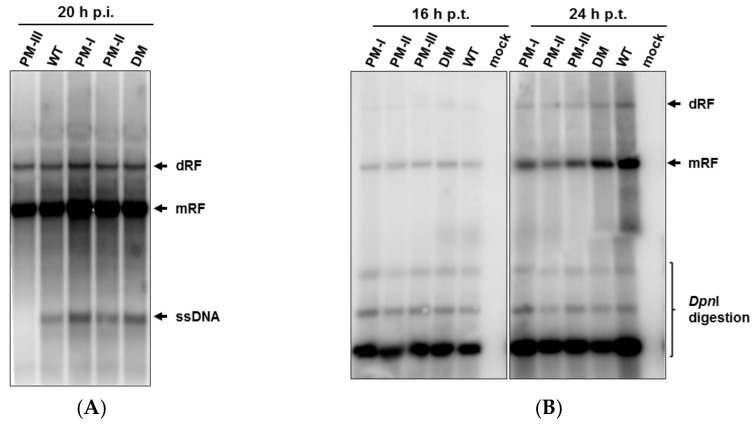
Accumulation of viral DNA replicative forms in infected or transfected NB-324k cells with the mutants. (**A**) Accumulation of DNA replicative forms in NB-324k cells infected with wt H-1PV and derived mutants. NB-324K cells (1.6 × 10^6^) were infected (MOI, 1 PFU/cell) with wt H-1PV, H1-PM-I, -II, -III, and –DM and further incubated in the presence of neutralizing antibodies. At 20 h post-infection, cells were harvested and viral DNA replicative forms were purified from cell lysates, separated by agarose gel electrophoresis, and subjected to Southern blotting. The bands corresponding to single-stranded, monomeric, and dimeric replicative forms of viral DNA are indicated as ssDNA, mRF, and dRF, respectively (**A**); (**B**) DNA replicative forms in NB-324k cells transfected with the plasmids pH1 (wt) or mutant derivatives. NB-324k cells (1 × 10^6^ cells) were transfected with 6 µg of pH1, pH1-PM-I, pH1-PM-II, pH1-PM-III, and pH1-DM and further incubated with neutralizing antibodies. Cells were harvested at indicated times post-transfection and viral DNA replicative forms purified from cell lysates were DpnI digested and analyzed by Southern blotting.

The enhanced yields of infectious particles and accumulated levels of viral proteins after infection with H1-PM-I, H1-PM-II, and H1-DM prompted us to determine which steps of the viral cycle were expected to be stimulated (or limited, H1-PM-III) in these mutants. For this purpose, the accumulation of viral DNA replicative forms was analyzed after infection (3PFU/cell) of NB-324k cells with either H-1PV or the mutants in the presence of neutralizing antibodies. Viral DNA replicative forms were extracted 20 h post-infection and subjected to Southern blot analysis (Figure 6A). The infection of NB-324k cells with H1-PM-I, H1PM-II, and H1-DM sustained slightly higher amounts of viral replicative forms (RF) than with wt H-1PV as shown by the signal intensities of the monomeric (mRF) and dimeric replicative forms (dRF) (Figure 6A). The higher levels of the progeny viral single-stranded DNA (ssDNA), which accumulated during a single viral cycle of infection with the mutants, was more striking (Figure 6A). The infection with H1-PM-III gave rise to lower levels of viral DNA replicative forms and ssDNA molecules were hardly detectable. Since striking differences were observed in the accumulation of the capsid proteins, while NS1 levels were similar to wt- and H1-PM-III-infected cells, we hypothesized that the low levels of ssDNA resulted from their reduced capsid production, preventing ssDNA displacement at the time of encapsidation. The viral DNA replicative forms were also analyzed in the absence of earlier steps of the viral cycle. For this, viral DNA molecules were extracted at 16 h and 20 h post-transfection of NB-324k cells with the plasmids pH1-PM-I, pH1-PM-II, pH1-PM-III, pH1-DM, and pH1, subjected to DpnI digestion, and analyzed by Southern blot assays. No increase in the accumulation of viral mRF and dRF molecules was observed among the samples transfected with the different plasmids compared with wt pH1 (Figure 6B). Consistent with the conclusion drawn above, this suggests that at least some steps of the viral cycle preceding the viral DNA replication—i.e., virus entry, transport to the nucleus, and/or uncoating—are likely to be stimulated in the fitness mutants.

### 3.5. No Increased Accumulation of the Viral Transcripts after Infection with the Mutants Compared with Wild-Type H-1PV 

**Figure 7:**
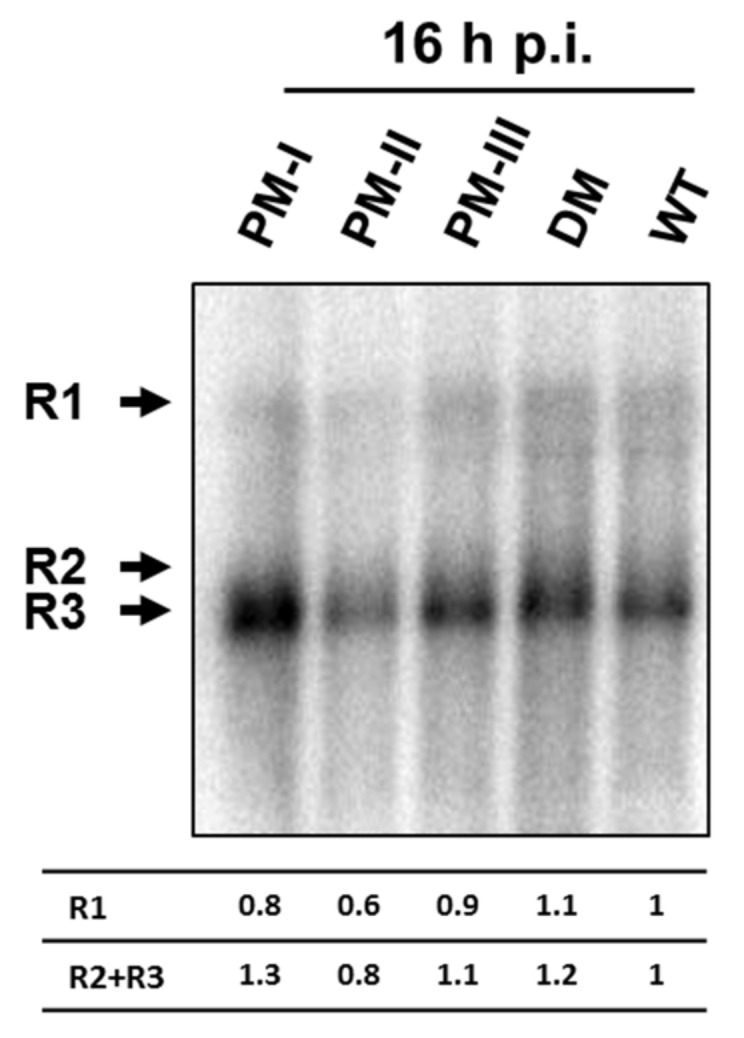
Accumulation of viral transcripts in infected NB-324k cells. NB-324k cell cultures infected (MOI of 3 PFU/cell) were incubated for 16 h and processed for Northern blotting analysis. The positions of viral R1, R2, and R3 mRNAs are indicated with arrows.

The nucleotide change(s) in the H-1PV genome of the mutants analyzed in this study results in amino acid modification(s) affecting either both NS1 and NS2 or NS2 alone (see Table 1 and Figure S1). A greater accumulation of NS2-encoding R2 transcripts and higher levels of NS2 proteins were reported for Del H-1PV, a fitness mutant containing an in-frame deletion in the NS coding sequence [21,22]. This prompted us to analyze the levels of viral transcripts R1, R2, and R3 coding for NS1, NS2, and VP proteins, respectively, after infection with wt H-1PV and the various mutants. This experiment was designed to analyze the viral transcripts in the absence of possible effects of the mutations on earlier steps of the viral cycle. For this purpose, NB-324k cells were infected with the same amounts of infectious particles of H-1PV, H1-PM-I, H1-PM-II, H1PM-III, and H1-DM (MOI = 3PFU/cell) and the viral transcripts were analyzed by Northern blotting of total RNA extracted 16 and 24 h post-infection. As seen in Figure 7, at 16 h post infection the levels of the viral transcripts, in particular R2, did not show significant differences between the mutants and H-1PV. This indicates that transcription and/or stability of the viral RNA were not modified by the mutations present in H1-PM-I, H1-PM-II, H1-DM, and H1-PM-III. It is worth noting that the accumulation of R3 transcripts (encoding the capsid proteins) after infection with H1-PM-III was similar to those of the control (H-1PV), suggesting that (a) post-transcriptional event(s) of the viral cycle is (are) responsible for the low levels of VP expressed after infection with this mutant.

### 3.6. Cis-Acting Effects of C2193A Mutation in H1-PM-III on Capsid Translation 

As shown above, the accumulation of viral capsids after infection with H1-PM-III was strikingly reduced compared with the wild-type control although the levels of the viral RNAs encoding the capsid proteins (R3) were not reduced. The 5′ untranslated region (5′-UTR) of R3 transcripts encoding VP1 and VP2 proteins overlap with sequences coding for the C-terminal domain of the NS2 protein. We next wanted to determine whether the NS2 mutation in H1-PM-III (L153M) per se was responsible for the poor capsid production with this mutant; for example, whether post-translational modification(s) of the capsids was making them unstable or whether this was due to a *cis*-effect of the mutation on the translation of the viral capsid RNA. Therefore, split vectors consisting of pP4-NS and pP38-VP expression plasmids were created from pH1 and pH1-PM-III, respectively, transfected into NB-324k cells, and VP production analyzed in protein extracts 24 and 48 h post-transfection by immunoblotting. Co-transfection of pP38-VP (wt) with pP4-NS-PM-III (expressing NS2-L153M protein) yielded levels of VP1 and VP2 similar to those with pP4-NS (wt). In contrast, the levels of VP1/2 were strongly reduced after co-transfection with pP4-NS (wt) and pP38-VP (PM-III) (Figure S2). This indicates that the C2193A mutation most likely affects in *cis* the expression of the capsid proteins independently of NS2-L153M. The reduced protein accumulation by C2193A mutation is independent of the sequence to be translated since this effect was also observed when VP was replaced by the Gaussia luciferase reporter (Figure S2).

### 3.7. Improved Virus Binding and Cellular Uptake of the Fitness Mutants

**Figure 8:**
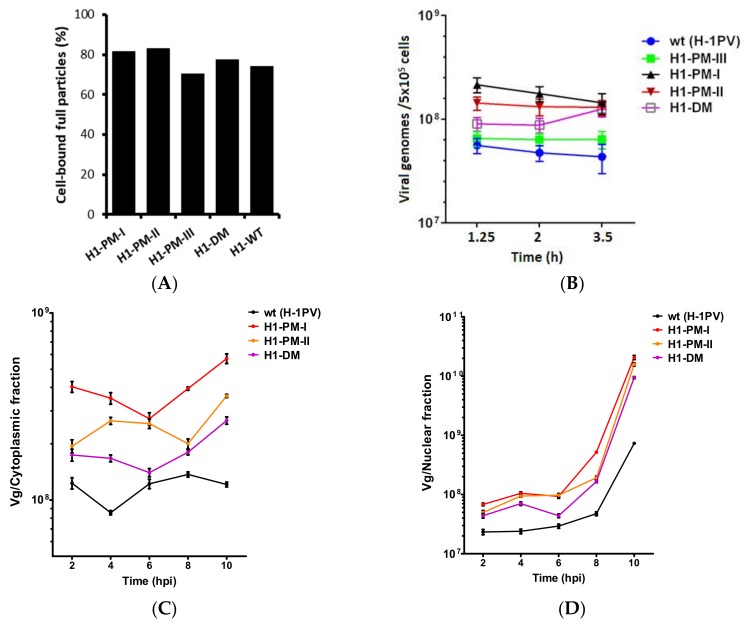
Comparison of cell binding (**A**), uptake (**B**); and nuclear import (**C**,**D**) of wt H-1PV particles and of H1-derived mutants. (**A**) NB-324K cells (5 × 10^5^) were infected with wt (H-1PV) or H1-PM-III, H1-PM-I, H1-PM-II, and H1-DM mutants at MOI of 2225 Vg/cell at 4 °C for 1 h. The amount of cell-bound full particles was quantified by qPCR and expressed as the percentage of total (cell-bound and supernatant) viral genome; (**B**) NB-324k cells (5 × 10^5^) were infected at 4 °C for 1 h with wt H-1PV or the mutants and further incubated at 37 °C. Cells were harvested at the indicated times of infection and treated with trypsin-EDTA. Viral genomes recovered from cell lysates were quantified by qPCR and given in Vg (**C**,**D**). Kinetics of cytoplasmic/nuclear distribution of wt and mutant viruses. NB-324K cells (5 × 10^5^) were infected and further incubated as for (**B**). At indicated times of infection, viral particles were isolated from cytoplasmic (**C**) and nuclear (**D**) fractions and the amount of viral genome determined by qPCR. Mean ± SD of two independent experiments.

Since viral DNA replication was stimulated after infection with H1-PM-I, H1-PM-II, and H1-DM, but not after transfection, we next wanted to determine whether the increased infectivity of these mutants correlated with a modification of the capsids, resulting in more efficient early steps of the virus cycle, in particular the binding and cell internalization of viral particles. The binding of virus particles was measured by incubating the cells at 4 °C (to prevent cell virus entry) and quantifying the genomes of free and cell-bound particles by qPCR. As indicated in Figure 8A, the fraction of full particles bound to the cells was slightly but reproducibly higher with H1-PM-I, H1-PM-II, and H1-DM than with wt H-1PV. The virus internalization was determined at various times of infection by qPCR of the intracellular viral genomes after removing the plasma membrane-bound particles with trypsin-EDTA treatment. In agreement with the binding assays, slightly higher amounts (2- to 3-fold higher) of viral genome was recovered from the cell lysates after 2 h of infection with the mutants compared with wt H-1PV (Figure 8B). Our results thus showed that with the exception of H1-PM-III, the binding and cell uptake are more efficient with the mutants, supporting the hypothesis of a modification in the viral capsids that may facilitate the cellular entry of the virions. Following their internalization, the virus particles are translocated to the nucleus where the viral DNA replication, capsid assembly, and genome packaging will occur and eventually produce progeny virions. To analyze some of these downstream steps, intracellular viral particles were next determined by qPCR of viral genomes in cytoplasmic and nuclear fractions of NB-324k cells infected with wt H-1PV or the mutants at 2 h up to 10 h of infection. In agreement with the virus uptake, more viral genomes were recovered from both cytoplasmic (Figure 8C) and nuclear (Figure 8D) fractions after infection with the mutants. However, the fraction of incoming particles (about 20%) that were recovered from the nuclear fraction was similar between the mutants and wt H-1PV, suggesting that the nuclear import of the mutants was not stimulated. In the nucleus, the levels of viral DNA increased dramatically about 6–8 h after infection, which can be assigned to the initiation of viral DNA replication. This resulted in a 10-fold increase in viral DNA accumulation with the mutants compared with wt at 10 h after infection (Figure 8D), in agreement with the enhanced accumulation of viral DNA replicative forms observed by infection with these mutants.

Overall, the decreased P/I ratio and more efficient binding and cellular uptake of the mutants suggest that post-translational modification(s) of the viral capsids are most likely responsible for the fitness phenotype of H1-PM-I, H1-PM-II, and H1-DM. Therefore, the phosphorylation pattern of the capsid proteins encoded by the mutants deserves investigation. The mutants express modified NS2 proteins: NS2-L103P (H1-PM-I), NS2-K96E (H1-PM-II), or both (H1-DM). The mutations affect also NS1 in H1-PM-I and H1-DM but not in H1-PM-II. Altogether, our results show that NS2-K96E increases the fitness of H-1PV and suggests that NS2-L103P also plays a role in this phenotype. Whether the modification in NS1 (Y595H) may also contribute to the fitness phenotype remains elusive.

## 4. Discussion

We previously reported that an in-frame 114-nucleotide deletion in the NS coding sequence of a mutant derived from H-1PV, Del H-1PV, showed key benefits for the fitness of this virus. In the present study, the sequence deleted in Del H-1PV was further investigated. To this end, either one (PM-I, PM-II, and PM-III) or two (DM; combining PM-I and PM-II) single nucleotide changes were introduced into the infectious molecular clone of H-1PV and the corresponding virus stocks were produced and analyzed. Because of the overlapping sequences of NS1 and NS2, the mutations affected either NS2 (H1-PM-II, H1-PM-III) or both NS1 and NS2 proteins (H1-PM-I, H1-DM). Three of the mutations that were introduced in H-1PV (H1-PM-I, H1-PM-II and H1-DM) clustered in the deleted region of Del H-1PV and enhanced the fitness of H-1PV. Indeed, the mutants displayed a large plaque phenotype and yielded titers of progeny particles with improved infectivity, as indicated by a lower particle-to-infectivity ratio (P/I) compared to wt H-1PV. Our experimental work did not reveal an accelerated nuclear egress of progeny H-1PV mutants (Figure S3), as it was reported for Del H-1PV [22], suggesting that the NS2-CRM1 interaction is not stronger in human cells infected with H1-PM-I, H1-PM-II, or H1-DM. It is generally accepted that protoparvoviruses, similar to other non-enveloped viruses, are released as a lytic burst, occurring at the end of infection. However, there is increasing evidence of an active pre-lytic egress of mature progeny [32]. It was shown that some post-translational modifications of the MVM viral capsids, in particular the phosphorylation of specific VP2 amino acids, play a key role in the maturation of progeny virions [33]. An active pre-lytic nuclear export of progeny virions was recently confirmed by Wolfisberg et al. [34] who identified two populations of MVM full particles with distinct surface charges in the nucleus. They showed that late capsid maturation involving further phosphorylation of surface residues was required for nuclear export, while these surface residues were dephosphorylated on incoming capsids [34]. However, since no direct interaction between NS2 and the capsids has been demonstrated so far, this activity of NS2 is likely to be indirect. In this study, we observed enhanced binding and uptake of H1-PM-I, H1-PM-II, and H1-DM. One can hypothesize that the NS2 amino acid residues modified in these mutants may induce the phosphorylation or even dephosphorylation of specific VP amino acids, thereby facilitating virus entry into subsequently infected cells. 

In contrast to MVMi, PM-III mutation, which affects the NS2 C-terminus (NS2 L153M) at a position downstream of the sequence deleted in Del H-1PV, impaired the virus replication and spreading. It is worth mentioning that the NS2 C-terminal coding sequence overlaps the 5′-UTR region of R3 viral mRNA encoding the capsid proteins. In H1-PM-III, the C2193A mutation generates a putative translation initiation codon and a small open reading frame. Whether this mutation interferes with R3 translation in the context of H-1PV remains to be determined. 

### Key Benefits of PM-I, -II, and –DM for H-1PV Fitness and Transduction Efficiency of H-1PV-Based Vectors

In the context of the H-1PV genome, this study shows, as also previously seen for Del H-1PV, key benefits of H1-PM-I, H1-PM-II, and H1-DM mutations with regard to the fitness of the virus stocks produced. This shows that the phenotype exhibited by Del H-1PV can be at least partly recapitulated by the nucleotide changes introduced in the NS coding sequence of H1-PM-I, H1-PM-II and H1-DM. 

Our results also indicate key benefits of the mutations and of the 114-nt deletion for the transduction efficacy of H-1PV-based vectors. However, the transduction efficiency depends on the tissue origin, as illustrated by the colon carcinoma cells HCT116 for which the highest transduced luciferase levels were obtained after infection with H1-DM; the other vectors were only slightly more efficient than the wild type or even less efficient, as for Del H-1PV. Although virus uptake and transport were not analyzed with the vectors, one can speculate about the viral capsids, presumably modified by the mutations, allowing higher amounts of internalized viral particles and, consequently, higher transduction efficiency. Parvoviral vectors fail to produce progeny viruses upon infection because the capsid-encoding region is replaced with a therapeutic or a marker transgene. The recombinant parvoviruses are produced by co-transfection with a helper plasmid providing the capsid genes. This indicates that NS2 (L103P) and/or NS1 (Y595H) and NS2 (K96E) act in *trans* on viral capsids. 

In addition to NS2 (L103P), the mutations in H1-PM-I and H1-DM also modify NS1, yet the contribution of the latter to the fitness of these viruses is still elusive. Nevertheless, NS2 plays a major role in this phenotype as shown by the fitness displayed by H1-PM-II where only NS2 (K96E), but not NS1, is modified. Whether both NS2 mutations cooperate to the fitness phenotype of H1-DM or NS1 (Y595H) and NS2 (K96E) is questionable. It is worth mentioning that in another study, an NS1 mutant of MVMp generated by site-directed mutagenesis (MVMp588A) [35] was described to give larger plaques and higher virus spreading than wild type in infected mouse A9 cultures. Interestingly, the mutation introduced in the viral genome also made an amino acid change in the small NS2 protein, affecting the leucine residue at the same position (namely, K96S) as for H1-PM-II. This points to the importance of the NS2 leucine residue at position 96, whose removal (Del H-1PV) or substitution (H1-PM-II and MVM) generates mutants with an enhanced capacity for propagation. Comparing the phosphorylation pattern of the capsids and their stability expressed by the mutants and the wild-type viruses might help to decipher the molecular mechanisms leading to the production of mature viruses and to shed light on NS2 functions. 

Recently, the first phase I/IIa clinical trial in which wild-type H-1PV was administered to recurrent glioblastoma patients [24] has provided promising results allowing further H-1PV clinical investigation, not only in glioblastoma patients but also in patients with metastatic pancreatic cancer [36]. However, the efficacy of H-1PV might be limited by the fact that the rodent protoparvovirus does not usually propagate in human tumor cells and, consequently, there is a need to optimize such viral therapeutics. Future steps in the validation of H-1PV treatment include adapting the virus for enhanced production, combining it with other cancer therapeutics, or arming H-1PV-based vectors with therapeutic transgenes. The mutants described in this study show that H-1PV and vector derivatives can be improved regarding their production and infectivity and may provide alternative tools for their application in cancers where wild-type H-1PV is less efficient.

## Figures and Tables

**Table 1:** Mutations introduced into the H-1PV genome and the corresponding modified amino acids in the NS proteins

Virus	Mutation(s)	Modified Protein(s)	Modified Amino Acid(s)
H-1-PM-I	T2044C	NS1/NS2	Tyr595His/Leu103Pro
H-1-PM-II	A2022G	NS2	Lys96Glu
H-1-DM	T2044C, A2022G	NS1/NS2	Tyr595His/Leu103Pro, Lys96Glu
H-1-PM-III	C2193A	NS2	Leu153Met
Del-H-1PV	Δnt2022-2135	NS1/NS2	38 aa deleted

## Supplementary Materials

The following are available online at http://www.mdpi.com/1999-4915/10/4/150/s1, Figure S1: Modified amino-acids following the introduction of specific point-mutations in the infectious molecular clone of H-1PV. Figure S2: Cis-effects of PM-III mutation (C193A) on P38-driven gene expression. Figure S3: Cytoplasmic/nuclear distribution of progeny viruses.
