# Supplementary material for: Mutations in the Non-Structural Protein-Coding Sequence of Protoparvovirus H-1PV Enhance the Fitness of the Virus and Show Key Benefits Regarding the Transduction Efficiency of Derived Vectors
Source: Viruses. 2018 Mar 27;10(4):150. doi: 10.3390/v10040150 (PMC5923444; doi:10.3390/v10040150)
Supplement: Supplementary file 1 [file viruses-10-00150-s001.zip › viruses-271463-supplementary.pdf]

Supplementary figures

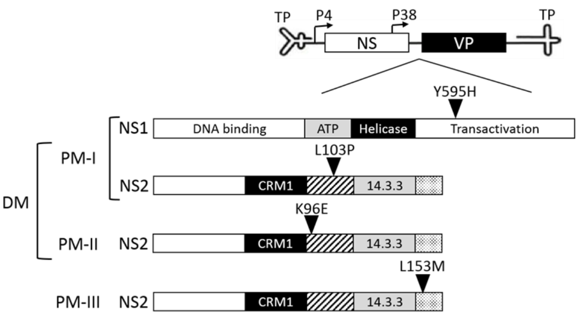

Figure S1. Modified amino-acids following the introduction of specific point-mutations in the infectious molecular clone of H-1PV. Scheme highlighting the position, within the NS1/NS2 functional domain(s), of the modified amino acids.

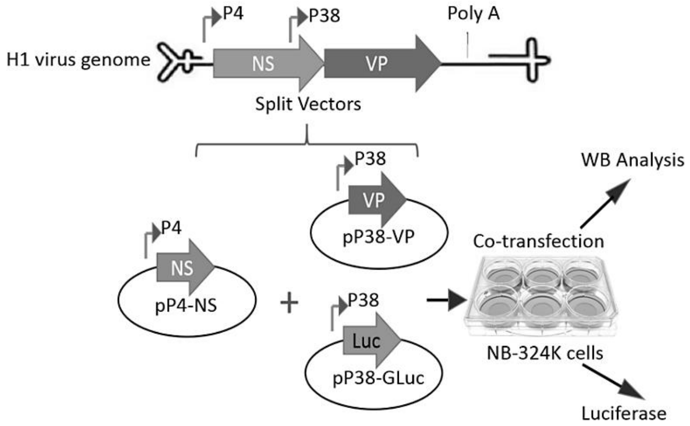

(**A**)

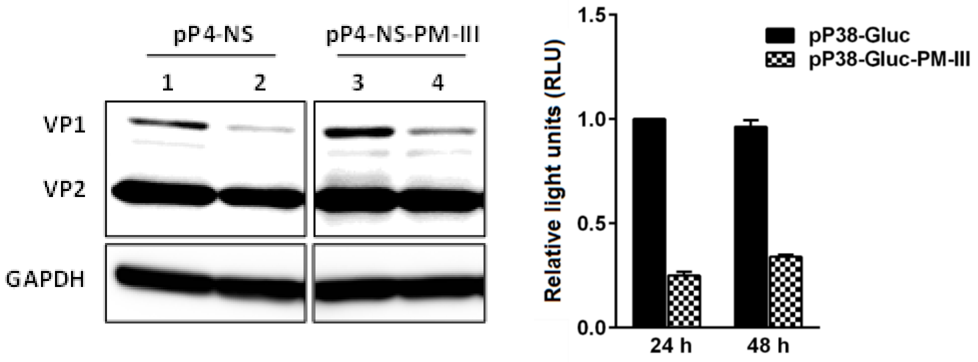

(**B**) (**C**)

Figure S2. Cis-effects of PM-III mutation (C193A) on P38-driven gene expression. (**A**) Scheme of split vectors derived from wt pH1 (pP4-NS, pP38-VP, pP38-Gluc), and from pH1-PM-III (pP4-NS-PM-III, pP38-VP-PM-III, pP38-Gluc-PM-III). (**B**) 0.5 μg of plasmids pP4-NS or pP4-NS-PM-III were co-transfected with 2 μg of either pP38-VP or pP38-VP-PM-III into human NB-234K cells ($2.7x10^5$). 24 h

after transfection, capsid proteins were analyzed from cell extracts by Western blotting. Cellular GAPDH was used for loading control Signal intensities of the VP proteins were quantified and normalized to corresponding wt VP referred to as 1. Lanes 1,3: cell lysates from co-transfection with pP38-VP, lanes 2,4: co-transfection with pP38-VP-PM-III. (**C**) Cis-effects of PM-III mutation on reporter gene expression. NB-324K cells (1x10^5) were co-transfected with 195 ng of wt (pP4-NS) and 780 ng of pP38-Gluc or pP38-Gluc-PM-III and 25 ng of pCMV-Cyp.luc expressing Cypridina luciferase as normalizing reporter. Gaussia and Cypridina activities were measured in the culture medium at 24h and 48 h after transfection. Gaussia luciferase values were normalized to those of Cypridina luciferase and expressed as relative light units (RLU) to pP38-Gluc (referred to as 1). Mean ± SD of three independent experiments.

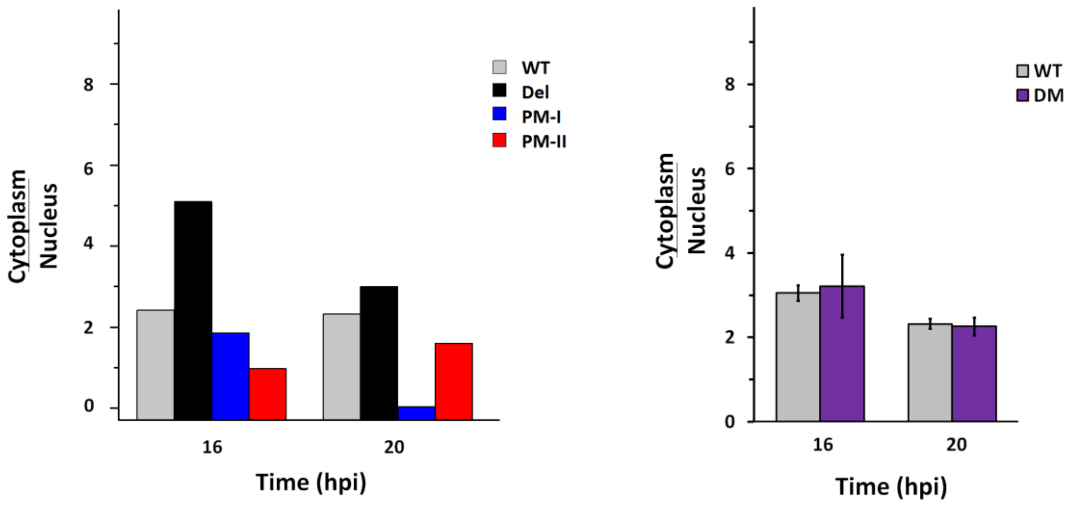

(**A**) (**B**)

Figure S3. Cytoplasmic/nuclear distribution of progeny viruses. NB-324k cells were infected with H1-PM-I, H1-PM-II, Del H-1PV (**A**) or H-1PV and H1-DM (**B**) (MOI=1 PFU/cell), and nuclear/cytoplasmic fractions were performed 16h and 20h post-infection in the presence of neutralizing antibodies. Infectious particles were quantified by plaque assays in both fractions. The ratio of infectious virus recovered from the cytoplasmic and nuclear fractions is shown.
